# Prediction of subjective well-being level in residents of Dali City: Where modern tourism meets traditional ethnic culture

**DOI:** 10.1371/journal.pone.0332625

**Published:** 2025-09-26

**Authors:** Ya Shi, Jingjing Zhu, Lin Fu, Keni Wu, Yong Zhao, Lingbin Ji, Yuanyuan Lan, Jinyong Wang, Xianglong Xu

**Affiliations:** 1 School of Public Health, Chongqing Medical University, Chongqing, China; 2 Research Center for Medicine and Social Development, Chongqing Medical University, Chongqing, China; 3 Research Center for Public Health Security, Chongqing Medical University, Chongqing, China; 4 Nutrition Innovation Platform-Sichuan and Chongqing, School of Public Health, Chongqing Medical University, Chongqing, China; 5 Department of Preventive Medicine, School of Public Health, Chongqing Medical and Pharmaceutical College, Chongqing, China; 6 School of Public Health, Shanghai University of Traditional Chinese Medicine, Shanghai, China; 7 Finance and Planning Department, Dali University, Dali, China; 8 School of Public Health, Dali University, Dali, China; Northeastern University (Shenyang China), CHINA

## Abstract

**Objective:**

Dali is a city rich in tourism resources and cultural heritage, where residents’ subjective well-being (SWB) varies in response to the dynamics of local tourism culture. Few studies have examined the distribution of SWB levels and their influencing factors in areas where modern tourism economies and traditional cultures coexist. The study aims to explore the relationship between multiple variables and SWB, and rank the importance of key well-being factors.

**Methods:**

This study employed a convenience sampling method to survey permanent residents of Dali City, resulting in a final dataset of 483 valid samples. Our study selected a wide range of predictors, including sociodemographic characteristics, leisure activities, social class identification, and preferences in socialization interaction patterns. Eight common ML algorithms were utilized to construct prediction models. The model’s performance was evaluated using the area under the curve (AUC) metric. Generalized additive models (GAMs) were used in sensitivity analyses to assess potential nonlinear relationships between predictors and SWB.

**Results:**

The probability of high SWB in Dali City was 48.9%. RF demonstrated the highest predictive accuracy (AUC = 0.82). By ranking the importance of variables in the best model RF, we obtain the top five predictors of SWB as: frequency of health issues affecting daily activities, family economic status, age, income, and weekly family face-to-face communication. GAMs explained 55.2% of the variance in SWB (R^2^ = 0.552, N = 483). Fewer health issues affecting daily life were strongly associated with higher SWB (B = 4.83–6.39, *p* < 0.001). Better family economic status (B = 1.37–2.24, *p* < 0.001) and greater trust in society (B = 0.92–1.39, *p* < 0.01) also predicted higher SWB. Age showed a positive association with SWB scores (EDF = 1.00, *p* < 0.001).

**Conclusions:**

This study shifts the focus from economic outcomes to residents’ SWB in a culturally diverse tourism setting. Using machine learning and GAMs, health issues emerged as the strongest predictors of SWB. Findings support health-oriented tourism strategies and highlight the need to integrate socio-cultural factors into sustainable tourism planning.

## Introduction

Well-being has received increasing research attention across the world in recent years [1,2]. Well-being refers to living a life filled with meaning, purpose, and self-realization [3]. Among the various aspects of well-being, subjective well-being (SWB) is the most widely studied [4,5]. Data has shown a consistent decline in life satisfaction, domain satisfaction, and happiness among US adolescents since 2010, and the rates of depression and suicide ideation have increased [6]. Comparable worrying trends are also observed in Europe [7]. The SWB of Chinese residents significantly varied across different regions [8] and has declined over the past two decades [9]. Critically, SWB is not merely a psychological metric but also a predictor of individual health (e.g., all-cause mortality) and social development (e.g., economic level) [10].

While the role of SWB has been extensively examined within the domains of individual, family, and social policy [6,9,11–14], important gaps persist in understanding its determinants in culturally distinct and tourism-oriented regions. Prior research has predominantly focused on Western populations or urban centers in China, with comparatively limited empirical attention given to areas such as Dali City multi-ethnic hub in Yunnan Province. Dali City exhibits considerable tourism development potential, with approximately 75% of its land area deemed suitable for cultural-tourism initiatives [15], and it reported tourism revenues of 78.27 billion yuan, ranking second in Yunnan Province [16]. Its distinctive natural environment and favourable climate are likely to contribute not only to economic growth but also to the enhancement of residents’ subjective well-being [17–19]. Moreover, Dali City is a melting pot of diverse ethnic groups, including the Bai, Lisu, and Achang [16]. The religious culture of the Bai ethnic group in Dali revolves around the worship of the ‘Ben Zhu’. Additionally, the pilgrimage to honor the ‘Ben Zhu’ serves as a significant source of SWB for residents [20]. This aligns with conclusions drawn in studies on the role of religion among Tibetan people in China [21]. Tourism development can exert multifaceted influences on residents’ well-being, extending beyond economic outcomes to encompass cultural and environmental dimensions [22]. However, excessive tourism exploitation has been associated with declines in residents’ quality of life [23]. In this context, SWB may serve as a critical indicator for assessing the integrity and sustainability of local socio-cultural systems. As active participants in tourism development, residents are particularly susceptible to the dynamics of the tourism market. For intangible cultural heritage (ICH) tourism, practitioners must account for residents’ cultural identity to mitigate the negative impacts of tourism on their attitudes and behaviors, thereby fostering the continuity of local culture while promoting sustainable tourism development [24]. Previous research has demonstrated that strengthening tourists’ cultural identity in ICH destinations can enhance ethnic identity, increase participation in religious activities, and facilitate resident-tourist value co-creation, ultimately improving life satisfaction [25]. Dali’s unique tourism-driven economic growth, multi-ethnic cultural integration, and religious practices have combined to create an environment that may differ from the mechanisms influencing SWB in homogeneous urban environments.

To date, research on SWB in culturally distinctive and tourism-driven regions remains limited, particularly concerning the complex interplay of socio-demographic, cultural, and contextual factors. These settings often involve unique patterns of well-being shaped by local traditions, ethnic diversity, and tourism-related transformations. However, such multidimensional influences have rarely been examined using advanced analytical approaches. Machine learning (ML) presents a promising avenue for capturing these nuanced relationships and enhancing our understanding of SWB in these underrepresented contexts. ML models outperform traditional logistic regression by handling complex nonlinear relationships, interactions, and multicollinearity [26,27]. ML algorithms do not need to rely on statistical inference or assumptions, and are self-optimizing and adaptive, thus providing more accurate and flexible tools for efficient risk prediction [27,28]. In addition, ML identifies important predictors that can provide personalized intervention strategies, which are critical for heterogeneous populations like the multi-ethnic residents of Dali. Although machine learning (ML) has been applied to SWB prediction in specific populations-such as Malaysian immigrants, African-American women with HIV/AIDS, and physicians use remains limited in geographically and culturally distinct regions [3,29,30]. In particular, there is a notable lack of research on predictive models for SWB in areas of southwestern China where religious beliefs and ethnic diversity play a significant role [21,31].

To address the research gap in both regional focus and methodological approach, we examined SWB in Dali City using a combination of machine learning and GAMs. As a culturally dynamic and tourism-driven region, Dali provides a unique context to identify complex, non-linear predictors of SWB. Our findings aim to guide targeted strategies for tourist service providers and facilitators, highlighting the importance of SWB-centered practices for the sustainable development of health-oriented tourism.

## Methods

### Study design and participants

In this cross-sectional study, we designed a questionnaire with reference to the China General Social Survey questionnaire (CGSS), taking into account the cultural background of the Dali region. We selected the resident population of Dali City with household registration, including the multi-ethnic populations of Bai, Han, and Yi, as the research subject, and distributed questionnaires to them through convenience sampling.

The sample size required for the study was estimated by the sample size calculation formula of the cross-sectional study as follows:


$$n=\frac{{Z_{\alpha }}^{\text{2}}\times pq}{d^{\text{2}}}$$

A previous study conducted in 2017 showed that the overall SWB rate among Chinese adults was 71% [32]. Therefore, we set *p* = 0.71, the d was 0.1*p*, and the confidence level was 0.95; a sample size of 158 could be calculated by PASS 15.0 (NCSS, LLC, Kaysville, Utah, USA). Considering the 20% non-response rate, the sample size was at least 190 participants. Ultimately, this study incorporated 483 valid samples, fulfilling the required sample size criteria. This study was approved by the Medical Ethics Committee of Dali University under ethical number MECDU-202108–20. Our study data included individuals (1) who have lived in Dali City for more than 1 year and are over 16 years old, (2) who can read and understand the questionnaire on their own or with the investigator’s explanation.

These participants possessed the capability to read and comprehend the questionnaire independently or with the assistance of the investigator’s explanations, and they were subsequently guided through the completion process to ensure the accuracy and completeness of their responses. The survey lasted 5 months, specifically from September 1st, 2021, to January 30th, 2022. In the quality control process, four investigators were involved, all of whom received specialized training. The investigators used quality control methods for cross-checking the questionnaires at the end of each day. The questionnaires were entered using a double-entry system.

### Predictors

Our study selected a wide range of predictors, including sociodemographic characteristics, outdoor leisure activities, social class identification, and socialization interaction pattern preference **(See Table 1**). Except for variables related to sociodemographic characteristics and socialisation interaction pattern preference, all other variables were measured using a 5-point Likert scale, with scores ranging from 1 to 5.

**Table 1:** Predictors of SWB.

Categories	Variables
Sociodemographic characteristics	gender, age, ethnic group, marital status, location of registered residence, education, father’s education, mother’s education, income, religious belief, number of properties owned, household car ownership, and investment.
Leisure activities	frequency of physical exercise, friend gatherings, relative gatherings, social activities, neighbour social recreational activities, and friend social recreational activities.
Social class identification	personal socioeconomic status, family economic status
Socialisation interaction pattern preference	weekly family face-to-face communication and weekly online communication with friends.
Others	frequency of health issues affecting daily activities, trust in society.

Sociodemographic characteristics include gender, age, ethnic group, marital status, location of registered residence, education, income, religious belief, number of properties owned, household car ownership, and investment..

Leisure activities include the frequency of physical exercise, friend gatherings, relative gatherings, social activities, neighbor social recreational activities, and friend social recreational activities. The Leisure Activities Scale, adapted from the CGSS questionnaire, utilizes a 5-point Likert-type scale ranging from Never (1) to Always (5).

Social identity theory posits that social identity is rooted in people’s subjective perception of their class identity [8]. This study measures social class identification through self-reported personal socioeconomic status and family economic status.

Socialization interaction pattern preference includes weekly family face-to-face communication and weekly online communication with friends. Socialization interaction pattern preferences were assessed by comparing the time spent on weekly face-to-face family communication versus weekly online communication with friends. Any non-zero time difference between the two was classified as a preference variation.

### Measurement of outcome

Subjective well-being (SWB) was assessed using a scale adapted from the Subjective Well-being section of the CGSS. It was measured through participants’ self-reported health status (e.g., ‘How would you rate your current health?’), life satisfaction (e.g., ‘How satisfied are you with your life overall?’), and the frequency of experiencing positive emotions (e.g., ‘How often do you feel happy or joyful?’). Each item was rated on a five-point Likert scale, and higher total scores indicated greater levels of SWB. We conducted reliability and validity tests on the 13-item questionnaire scales. The scale demonstrated reasonable internal consistency in the current sample, with a Cronbach’s alpha of 0.70. The Kaiser-Meyer-Olkin (KMO) measure of sampling adequacy was 0.71, suggesting that the data were appropriate for factor analysis and supporting the construct validity of the scale.

To improve interpretability for public health policymakers, SWB was dichotomized at the median into ‘high’ and ‘low’ groups in the machine learning analysis. This binary approach is commonly used in SWB research and helps maintain consistency with established analytical methods in the field [10].

### Statistical analysis

R version 4.1.3 and Stata software (version 18.0, Stata Corp, LLC) were used for data processing. The median (M) and interquartile range (IQR) were used for numerical data. Meanwhile, the composition ratio was used for categorical data. All statistical tests were executed using a two-sided approach, with statistical significance defined at *p* < 0.05.

### Machine learning

We used an 80%–20% split ratio to divide the dataset into training and test sets, and performed five-fold cross-validation on the training set. Eight common ML algorithms were built using the scikit-learn library in Python 3.8.12. These algorithms included logistic regression (LR), adaptive boosting classifier (AdaBoost), support vector machine (SVM), random forest (RF), Gaussian naive Bayes (GNB), gradient boosting machine (GBM), light gradient boosting decision machine (LGBM), and K nearest neighbor (KNN).

We chose those models because they have shown good performance in similar studies and are capable of handling complex datasets. LR is a simple supervised classifier for binary problems [26]. SVM excels in complex data structures for classification and regression [26]. RF combines multiple decision trees for stable, accurate predictions [26]. AdaBoost enhances weak learners iteratively for unbalanced data [33]. KNN is straightforward for small dataset classification and regression [34]. Naive Bayes assumes feature independence and performs well in applications such as text classification and clustering. GNB is a special case of NB, which assumes that the feature data obeys a Gaussian distribution (i.e., normal distribution) [34]. Light GBM is an algorithm based on gradient boosting trees that aims to improve prediction accuracy by stacking weak classifiers [35]. GBM iteratively trains weak classifiers for optimal performance [36]. These models can capture nonlinear relationships between variables, which is important in SWB research where the relationships may not be straightforward.

The model’s performance was evaluated using the area under the curve (AUC) metric, which represents the area under the receiver operating characteristic (ROC) curve. An AUC value ranging from 0.7 to 0.8 is considered acceptable, 0.8 to 0.9 is regarded as excellent, and any value exceeding 0.9 is categorized as outstanding [27].

Finally, based on the AUC results, we will use feature importance analysis in the best model to explain the relationship between variables, with scores showing the contribution of each variable to the prediction. This will be verified in combination with traditional statistical analysis.

### Sensitivity analyses

We used GAMs to examine potential nonlinear associations between predictors and SWB scores. GAMs extend generalized linear models by allowing flexible, curvilinear relationships and interactions between covariates [37,38]. Based on prior evidence of U-shaped associations with age, income, weekly face-to-face family communication, and online communication with friends, we applied penalized smoothing splines [39]. The effective degrees of freedom (EDF) quantify the shape of each association: an EDF of 1 suggests linearity, while values >1 indicate increasing nonlinearity [40].

Given 24 predictors across five domains (see Table 1), we built five domain-specific models (See Supplementary Table 1 in S1 File). Only predictors with significant associations in the final model are presented.

## Results

### Characteristics of the study data

Our study included a total of 483 participants, comprising 209 females and 274 males, with 203 from urban areas and 280 from rural areas. The median age was 44 years, with an interquartile range (IQR) of 37–50 years. The occurrence of a high level of SWB was 48.9%. The detailed sociodemographic data and other related information are illustrated in Table 2, Supplementary Tables 2 and 3 in S1 File.

**Table 2:** Cross-tabulation of study participants by gender.

Characteristic	Total, N = 483^1^	male, N = 274^1^	female, N = 209^1^	p-value^2^
**Age**	44 (37,50)	46 (39,51)	40 (33,48)	**<0.001**
**Ethnic group**				0.085
Han	225(46.6%)	137(50.0%)	88(42.1%)	
Others	258(53.4%)	137(50.0%)	121(57.9%)	
**Location of registered residence**				0.9
Rural	280(58.0%)	158(57.7%)	122(58.4%)	
Urban	203(42.0%)	116(42.3%)	87(41.6%)	
**Marital status**				0.12
Not married	28(5.8%)	10(3.6%)	18(8.6%)	
Married	427(88.4%)	249(90.9%)	178(85.2%)	
Divorced	16(3.3%)	8(2.9%)	8(3.8%)	
Widowed	12(2.5%)	7(2.6%)	5(2.4%)	
**Education**				0.077
Elementary school or lower	74(15.3%)	38(13.9%)	36(17.2%)	
Middle school	159(32.9%)	92(33.6%)	67(32.1%)	
High school or vocational school	176(36.4%)	110(40.1%)	66(31.6%)	
College or higher	74(15.3%)	34(12.4%)	40(19.1%)	
**Income**	65,000 (24,000,90,000)	70,000 (36,000,100,000)	53,000 (20,000,80,000)	**<0.001**
**SWB**				0.3
Low	247(51.1%)	135(49.3%)	112(53.6%)	
High	236(48.9%)	139(50.7%)	97(46.4%)	

^1^Categorical variables are presented as number of participants (%); numeric variables are presented as the Median (25%,75%)

^2^Pearson’s Chi-squared test; Wilcoxon rank sum test; Fisher’s exact test

### Prediction of stroke

**Fig 1:**
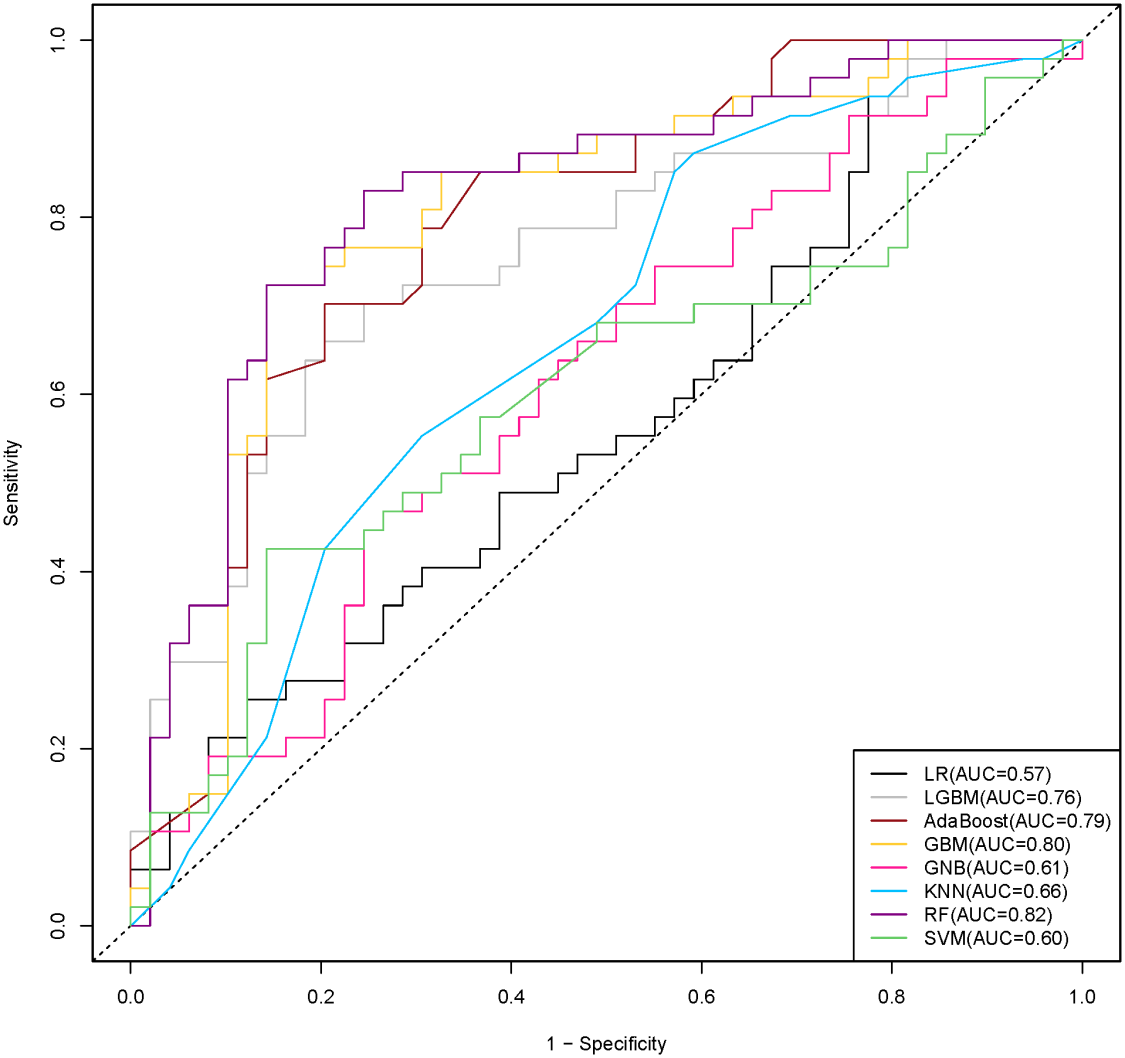
ROC curve performance of SWB prediction.

The ROC curves of the eight ML algorithms applied to the predictive models are depicted in **Fig 1**. RF demonstrated the highest predictive accuracy (AUC = 0.82), followed by GBM (AUC = 0.80), AdaBoost (AUC = 0.79), LGBM (AUC = 0.76), KNN (AUC = 0.66), GNB (AUC = 0.61), SVM (AUC = 0.60), and LR (AUC = 0.57).

#### Important predictors for SWB.

**Fig 2:**
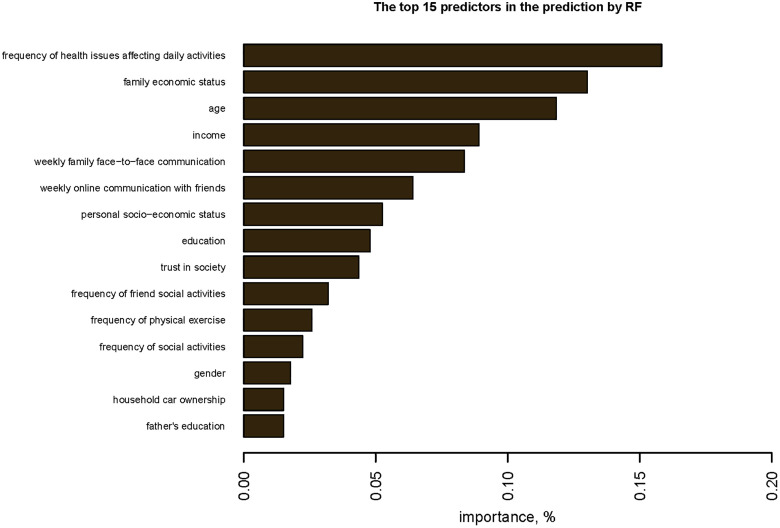
Top 15 predictors in the prediction of SWB by the best model.

By ranking the importance of the variables in the best model RF, we conclude that the fifteen most important variables in predicting SWB are as follows: frequency of health issues affecting daily activities, family economic status, age, income, weekly family face-to-face communication, weekly online communication with friends, personal socioeconomic status, education, trust in society, frequency of friend social activities, frequency of physical exercise, frequency of social activities, gender, household car ownership, and father’s education **(see Fig 2**). The higher the ranking, the more critical the role it plays in the predictive performance of the model.

#### Sensitivity analyses: the predictors associated with SWB using generalized additive models.

Final model explained 55.2% of the variance in SWB (R^2^ = 0.552, N = 483). SWB increased with fewer health issues affecting daily life. Compared to those always affected, participants reporting issues ‘sometimes’, ‘very few’, or ‘never’ had notably higher predicted SWB scores (B = 4.83–6.39, *P* < 0.001).

Better family economic status was positively associated with SWB. Individuals reporting ‘average’ to ‘well above average’ economic conditions had higher scores than those ‘well below average’ (B = 1.37–2.24, *P* < 0.001).

Greater trust in society also predicted higher SWB. Participants who ‘agreed’ or ‘strongly agreed’ had significantly elevated SWB compared to those who ‘strongly disagreed’ (B = 0.92 and 1.39; P < 0.01 and < 0.001, respectively). Age was positively associated with SWB in a near-linear form (EDF = 1.00, *P* < 0.001). (See Table 3 and Supplementary Fig 1 in S2 File).

**Table 3:** Factors associated with subjective well-being using generalized additive models analyses.

	subjective well-being (N = 483, R^2^ = 0.552)	
**Variables**	**B**	***P***
**Frequency of health issues affecting daily activities**		
Always	Reference Group	
Often	1.73	0.054
Sometimes	4.83	<0.001^***^
Very few	5.65	<0.001^***^
Never	6.39	<0.001^***^
**Family economic status**		
Well below average level	Reference Group	
Below average level	0.38	0.105
Average level	1.37	<0.001^***^
Above average level	1.41	<0.001^***^
Well above average level	2.24	<0.001^***^
**Trust in society**		
Strongly disagree	Reference Group	
Disagree more	0.07	0.831
Neutral	0.46	0.129
Agree	0.92	<0.01^**^
Strongly agree	1.39	<0.001^***^
**Age**	Smooth Curve, EDF = 1.00	<0.001^***^

**p < 0.01, ***p < 0.001

## Discussion

### Key findings

Our research is a machine-learning initiative for predicting SWB among Dali city residents. The results showed that the proportion of individuals with high SWB reached about half of the population in Dali city. RF was the optimal predictive model, achieving an AUC of 0.82 with the Dali data set. The most significant predictor of SWB level was the frequency of health issues affecting daily activities, followed by family economic status and age. Furthermore, we used GAMs to examine predictors of SWB and found that the frequency of health issues affecting daily life was the strongest factor, followed by economic status, social trust, and age.

### Compared with the evidence in the literature and research value

Relevant reviews indicate that AI and ML have significant potential to derive inferences and identify patterns in vast volumes of patient histories, medical images, and epidemiological statistics [41,42]. However, its application to the study of cultural well-being remains limited. The ML model developed by our study achieved a prediction performance of 82% for the SWB levels of Dali residents, higher than the traditional binary logistic analysis, thereby promoting research in this area. The model identifies key predictors that can be changed to enable targeted policy interventions in areas of minority cultures, to improve the well-being of individuals and communities.

Recent cross-sectional studies have found a close relationship between the current disease state or the overall physical health 1 year ago among adults with SWB, consistent with our results. Specifically, a physical condition with a low level of influence on daily life will have a higher level of SWB [43,44]. The favorable climate conditions in Dali, along with low air pollution levels and a reduced incidence of inflammatory chronic diseases, may contribute to the higher SWB levels observed in its residents [19,45]. Related studies have also shown that high levels of SWB have a corresponding benefit on the body’s recovery from disease and life expectancy [46,47], the two complement each other, promoting individual health.

SWB, a measure of one’s satisfaction with their current state of reality, is expected to fluctuate with the dynamics of age and gender. The researcher found that women in the general Swedish population reported lower well-being than men, and the elderly individuals (70–80 years of age) reported higher SWB than younger people [12]. This age difference may be related to the life events that people experience as they age. For example, major life changes such as retirement and widowhood often lead to simplification of social roles, and simpler lifestyles may be associated with higher levels of SWB [48]. This phenomenon observed in Dali is primarily because elderly residents in Dali tend to gather in their later years, engage in casual conversations, and increase their social interactions with one another. These social interactions are consistent with the factors found in the study to increase life satisfaction [49]. Furthermore, the ethnic dances and cultural activities in Dali provide more opportunities for physical exercise, and physical activity, especially outdoor recreation, is associated with higher levels of SWB [50,51].

The family economy, the foundation of a person’s existence, plays an important role in the development of his or her mental health. The population of European immigrants, which lacks a group of economically disadvantaged families, exhibits a smaller difference between their own SWB and that of the natives, suggesting that SWB is more stable when the family’s economic situation is moderately high [52]. Wang Peng scholars found that in China’s four northeastern provinces and ethnic minority regions, such as Inner Mongolia, Gansu, Ningxia, and Xinjiang, high-income levels will greatly contribute to the positive development of high levels of SWB [53]. This notion is consistent with the finding in this study that residents of the Dali ethnic minority area who are in the middle and above household economic statuses are positively correlated with the level of SWB. This is primarily attributed to the thriving tourism, cultural, and economic sectors in Dali, which have generated additional income for local residents, provided increased employment opportunities for the youth, and enhanced social recognition.

The relationship between religious and cultural beliefs and SWB in Dali city, as a gathering place for ethnic minorities, was also the focus of the study. Previous research has shown that religious behavior among rural residents in southern India enhances social cohesion and indirectly contributes to well-being [54]. However, we did not find the mutual influence of the presence or absence of religious beliefs on the level of SWB of Dali residents in this study. This situation may be due to the study area’s proximity to an urbanized area, where religious culture has been influenced. Dali, as a tourist city, has been constantly exposed to foreign cultures, influencing the local cultural ecology and the SWB of the residents.

### Strengths and limitations

In light of the findings, this study also has some limitations. First, the SWB in this study was derived from the subjects’ self-reports, positive emotions, and health status, which were specific responses at the time, and subjective bias was present. Although this approach offers insights directly from the participants, such susceptibilities might skew the data and affect the model’s performance. In addition, a certain level of Mandarin proficiency was required for inclusion in this study to minimize the survey’s information bias. Consequently, the sample excludes individuals who have lived in the local area for a long time, speak only the local language, and the scope of the study is limited to Dali City, yielding limited results. And another limitation of this study is that the internal consistency of the SWB scale, while close to the acceptable threshold (Cronbach’s alpha = 0.70), did not reach the conventional standard for good reliability. This may reflect constraints in the scale’s validity, particularly given the small number of items (About 10 items). However, as this measure represents an initial attempt to assess SWB in ethnic minority areas, and considering that a Cronbach’s alpha between 0.6 and 0.8 is generally acceptable for brief scales in exploratory studies [55]. Moreover, to align with common practice in tourism policy planning, we categorized SWB into ‘high’ and ‘low’ levels. While this may have reduced data granularity, sensitivity analyses using continuous SWB in GAMs produced consistent results, helping to address this limitation. Furthermore, since convenience sampling was used to collect data, statistical inferences may be limited in terms of generalizability in the statistical analysis section, and caution should be exercised when interpreting *p*-values. Lastly, although this study uses ML technology, it does not determine whether SWB is a cause or an effect of other risk factors. Further research is needed to unravel these complex interconnections.

Despite these limitations, the strengths of this study are manifold. Our study selected the Dali region, which has a strong religious culture. However, cultural intermingling may influence the prediction of SWB among residents and the analysis of influencing factors due to the development of the tourism economy. This work fundamentally differs from previous studies that primarily focused on traditional tourism economic outcomes or were conducted within closed religious and cultural contexts or among specific occupational groups [21,30]. Furthermore, both the machine learning and GAMs identified the frequency of health issues affecting daily life as the strongest predictor of individual SWB. The dominant role of health factors highlights the potential for health-oriented tourism development-such as wellness tourism, culturally rooted healing experiences, and rehabilitation retreats-which can enhance satisfaction for residents and contribute to responding to the call for national fitness activities and improving the well-being index [34]. Meanwhile, this study further confirms the important role of family economic status, trust in society for SWB in minority culture areas among different age groups. These findings highlight the importance of integrating socioeconomic conditions and social trust into tourism policy and community planning to promote residents’ mental health and ensure the sustainable development of minority cultural regions.

## Conclusion

This study is the first to apply a machine learning framework to examine how socioeconomic, health, and socio-cultural factors jointly shape the SWB of residents in multi-ethnic cultural tourism destinations such as Dali City. The analysis revealed that health-related variables-particularly the frequency of health issues affecting daily activities-were the strongest predictors of SWB, followed by family economic status, age, income, and social connectedness. These findings highlight the multidimensional nature of SWB, where material, health, and relational resources intersect within a unique cultural tourism context.

### Theoretical contributions

Our findings extend the tourism literature by introducing machine learning as an analytical tool for unpacking complex SWB determinants in culturally distinct, tourism-oriented regions. The results reinforce theories linking health status, economic resources, and social trust to well-being, while situating these relationships within a multi-ethnic cultural tourism environment. By demonstrating the salience of socio-cultural interaction (e.g., family communication) alongside material and health-related determinants, the study advances theoretical models that bridge social capital theory and tourism well-being research.

### Practical implications

The results offer actionable insights for market segmentation and personalized tourism service delivery. Differences in SWB by age group, family economic status, and health condition suggest that tourism providers should tailor products and experiences to demographic and psychosocial profiles. The strong influence of health-related factors points to the potential for health-oriented tourism strategies—such as wellness programs, active-aging initiatives, and community-based health promotion—that can simultaneously improve resident satisfaction and enhance visitor experiences.

### Cultural and social underpinnings

This study underscores the critical role of cultural identity and social cohesion in shaping perceptions and outcomes of tourism development. Higher trust in society and frequent meaningful interactions were positively linked to SWB, highlighting the socio-cultural fabric as both a driver and beneficiary of tourism. In destinations like Dali, where intangible cultural heritage and religious practices are deeply embedded in daily life, culturally sensitive policies that respect and integrate local traditions can foster resident–tourist value co-creation. Such approaches not only safeguard cultural continuity but also embed psychological sustainability into long-term tourism planning, ensuring that economic growth does not come at the expense of community well-being.

Ultimately, by elucidating the intertwined roles of health, economic security, and socio-cultural cohesion in shaping residents’ SWB, this study offers a transferable framework for evaluating and enhancing well-being in culturally diverse tourism destinations worldwide. Embedding these insights into tourism policy and practice can foster not only sustainable economic growth but also resilient, culturally vibrant communities.

## Supporting information

S1 File Supplementary Tables 1–3. (DOCX)

S2 File Supplementary Fig 1. (TIF)

## Abbreviations

AdaBoost: adaptive boosting classifier
AUC: the area under the receiver operating characteristic curve
CI: confidence interval
GBM: gradient boosting machine
GNB: gaussian naive Bayes
KNN: K Nearest Neighbours
LGBM: light gradient boosting decision machine
LR: logistic regression
ML: machine learning
OR: odds ratio
RF: random forest
ROC: receiver operating characteristic
SVM: support vector machines
SWB: Subjective well-being
