# Supplementary material for: Prediction of subjective well-being level in residents of Dali City: Where modern tourism meets traditional ethnic culture
Source: PLoS One. 2025 Sep 26;20(9):e0332625. doi: 10.1371/journal.pone.0332625 (PMC12469199; doi:10.1371/journal.pone.0332625)
Supplement: S1 File — (DOCX) [file pone.0332625.s001.docx]

| Response | Predictor |
| --- | --- |
| Model 1: SWB scores | Smooth(age),gender, ethnic group, marital status, location of registered residence, education, father's education, mother's education, and smooth(income) |
| Model 2: SWB scores | Significant predictors identified in Model 1+ personal socioeconomic status, family economic status |
| Model 3: SWB scores | Significant predictors identified in Model 2+ smooth(weekly family face-to-face communication) ,smooth(weekly online communication with friends) |
| Model 4: SWB scores | Significant predictors identified in Model 3+ frequency of physical exercise, friend gatherings, relative gatherings, social activities, neighbour social recreational activities, friend social recreational activities |
| Model 5: SWB scores | Significant predictors identified in Model 4+ religious belief, frequency of health issues affecting daily activities, trust in society, number of properties owned, household car ownership, investment |

***Supplementary Table 1 Five GAM models to explore associations between predictors and SWB scores***

Supplementary Table 2 Cross-tabulation of study participants by gender

| **Characteristic** | **N = 483**^1^ | **male**, N = 274^1^ | **female**, N = 209^1^ | | **p-value**^2^ |
| --- | --- | --- | --- | --- | --- |
| **Gender** |  |  |  | | - |
| Male | 274(56.7%) | - | - | |  |
| Female | 209(43.3%) | - | - | |  |
| **Ethnic group** |  |  |  | | 0.085 |
| Han | 225(46.6%) | 137(50.0%) | 88(42.1%) | |  |
| Others | 258(53.4%) | 137(50.0%) | 121(57.9%) | |  |
| **Location of registered residence** |  |  |  | | 0.9 |
| Rural | 280(58.0%) | 158(57.7%) | 122(58.4%) | |  |
| Urban | 203(42.0%) | 116(42.3%) | 87(41.6%) | |  |
| **Marital status** |  |  |  | | 0.12 |
| Not married | 28(5.8%) | 10(3.6%) | 18(8.6%) | |  |
| Married | 427(88.4%) | 249(90.9%) | 178(85.2%) | |  |
| Divorced | 16(3.3%) | 8(2.9%) | 8(3.8%) | |  |
| Widowed | 12(2.5%) | 7(2.6%) | 5(2.4%) | |  |
| **Education** |  |  |  | | 0.077 |
| Elementary school or lower | 74(15.3%) | 38(13.9%) | 36(17.2%) | |  |
| Middle school | 159(32.9%) | 92(33.6%) | 67(32.1%) | |  |
| High school or vocational school | 176(36.4%) | 110(40.1%) | 66(31.6%) | |  |
| College or higher | 74(15.3%) | 34(12.4%) | 40(19.1%) | |  |
| **Father's education** |  |  |  | | 0.2 |
| Elementary school or lower | 283(58.6%) | 166(60.6%) | 117(56.0%) | |  |
| Middle school | 159(32.9%) | 90(32.8%) | 69(33.0%) | |  |
| High school or vocational school | 39(8.1%) | 18(6.6%) | 21(10.0%) | |  |
| College or higher | 1(0.2%) | 0(0.0%) | 1(0.5%) | |  |
| Other | 1(0.2%) | 0(0.0%) | 1(0.5%) | |  |
| **Mother's education** |  |  |  | | 0.4 |
| Elementary school or lower | 346(71.6%) | 201(73.4%) | 145(69.4%) | |  |
| Middle school | 117(24.2%) | 63(23.0%) | 54(25.8%) | |  |
| College or higher | 2(0.4%) | 0(0.0%) | 2(1.0%) | |  |
| Other | 18(3.7%) | 10(3.6%) | 8(3.8%) | |  |
| **Income** | 65,000 (24,000,90,000) | 70,000 (36,000,100,000) | 53,000 (20,000,80,000) | | **<0.001** |
| **Frequency of health issues affecting daily activities** |  |  |  | | 0.5 |
| Always | 2(0.4%) | 2(0.7%) | 0(0.0%) | |  |
| Often | 15(3.1%) | 8(2.9%) | 7(3.3%) | |  |
| Sometimes | 171(35.4%) | 100(36.5%) | 71(34.0%) | |  |
| Rarely | 240(49.7%) | 129(47.1%) | 111(53.1%) | |  |
| Never | 55(11.4%) | 35(12.8%) | 20(9.6%) | |  |
| **Frequency of physical exercise** |  |  |  | | 0.5 |
| Never | 66(13.7%) | 40(14.6%) | 26(12.4%) | |  |
| A few times a year or less | 154(31.9%) | 93(33.9%) | 61(29.2%) | |  |
| A few times a month | 177(36.6%) | 91(33.2%) | 86(41.1%) | |  |
| Several times a week | 58(12.0%) | 34(12.4%) | 24(11.5%) | |  |
| Daily | 28(5.8%) | 16(5.8%) | 12(5.7%) | |  |
| **Frequency of friend gatherings** |  |  |  | | **0.005** |
| Never | 16(3.3%) | 3(1.1%) | 13(6.2%) | |  |
| A few times a year or less | 247(51.1%) | 141(51.5%) | 106(50.7%) | |  |
| A few times a month | 200(41.4%) | 119(43.4%) | 81(38.8%) | |  |
| Several times a week | 17(3.5%) | 11(4.0%) | 6(2.9%) | |  |
| Daily | 3(0.6%) | 0(0.0%) | 3(1.4%) | |  |
| **Frequency of relative gatherings** |  |  |  | | 0.2 |
| Never | 41(8.5%) | 22(8.0%) | 19(9.1%) | |  |
| A few times a year or less | 380(78.7%) | 217(79.2%) | 163(78.0%) | |  |
| A few times a month | 58(12.0%) | 35(12.8%) | 23(11.0%) | |  |
| Several times a week | 2(0.4%) | 0(0.0%) | 2(1.0%) | |  |
| Daily | 2(0.4%) | 0(0.0%) | 2(1.0%) | |  |
| **Frequency of social activities** |  |  |  | | **0.021** |
| Never | 6(1.2%) | 0(0.0%) | 6(2.9%) | |  |
| Rarely | 134(27.7%) | 70(25.5%) | 64(30.6%) | |  |
| Sometimes | 215(44.5%) | 127(46.4%) | 88(42.1%) | |  |
| Often | 115(23.8%) | 71(25.9%) | 44(21.1%) | |  |
| Always | 13(2.7%) | 6(2.2%) | 7(3.3%) | |  |
| **Frequency of neighbor social activities** |  |  |  | | **0.006** |
| Never | 21(4.3%) | 5(1.8%) | 16(7.7%) | |  |
| Rarely | 135(28.0%) | 75(27.4%) | 60(28.7%) | |  |
| Sometimes | 257(53.2%) | 160(58.4%) | 97(46.4%) | |  |
| Often | 55(11.4%) | 26(9.5%) | 29(13.9%) | |  |
| Always | 15(3.1%) | 8(2.9%) | 7(3.3%) | |  |
| **Frequency of friend social activities** |  |  |  | | **0.025** |
| Never | 7(1.4%) | 0(0.0%) | 7(3.3%) | |  |
| Rarely | 191(39.5%) | 112(40.9%) | 79(37.8%) | |  |
| Sometimes | 194(40.2%) | 107(39.1%) | 87(41.6%) | |  |
| Often | 81(16.8%) | 50(18.2%) | 31(14.8%) | |  |
| Always | 10(2.1%) | 5(1.8%) | 5(2.4%) | |  |
| **Trust in society** |  |  |  | | 0.8 |
| Strongly disagree | 19(3.9%) | 10(3.6%) | 9(4.3%) | |  |
| Comparatively disagree | 52(10.8%) | 28(10.2%) | 24(11.5%) | |  |
| Neutrality | 118(24.4%) | 63(23.0%) | 55(26.3%) | |  |
| Relatively agree | 266(55.1%) | 156(56.9%) | 110(52.6%) | |  |
| Couldn't agree more | 28(5.8%) | 17(6.2%) | 11(5.3%) | |  |
| **Personal socio-economic status** |  |  |  | | 0.7 |
| Lower class | 47(9.7%) | 29(10.6%) | 18(8.6%) | |  |
| Lower middle class | 118(24.4%) | 62(22.6%) | 56(26.8%) | |  |
| Middle-ranking | 242(50.1%) | 142(51.8%) | 100(47.8%) | |  |
| Upper-middle class | 70(14.5%) | 37(13.5%) | 33(15.8%) | |  |
| Upper class | 6(1.2%) | 4(1.5%) | 2(1.0%) | |  |
| **Family economic status** |  |  |  | | 0.14 |
| Well below average level | 37(7.7%) | 25(9.1%) | 12(5.7%) | |  |
| Below average level | 127(26.3%) | 64(23.4%) | 63(30.1%) | |  |
| Average level | 238(49.3%) | 140(51.1%) | 98(46.9%) | |  |
| Above average level | 74(15.3%) | 39(14.2%) | 35(16.7%) | |  |
| Well above average level | 7(1.4%) | 6(2.2%) | 1(0.5%) | |  |
| **Number of properties owned** |  |  |  | | **0.005** |
| 0 | 17(3.5%) | 6(2.2%) | 11(5.3%) | |  |
| 1 | 372(77.0%) | 202(73.7%) | 170(81.3%) | |  |
| 2 | 84(17.4%) | 61(22.3%) | 23(11.0%) | |  |
| 3 | 7(1.4%) | 4(1.5%) | 3(1.4%) | |  |
| 4 | 3(0.6%) | 1(0.4%) | 2(1.0%) | |  |
| **Household car ownership** |  |  |  | | 0.2 |
| Yes | 246(50.9%) | 147(53.6%) | 99(47.4%) | |  |
| No | 237(49.1%) | 127(46.4%) | 110(52.6%) | |  |
| **Family investment activities** |  |  |  | | 0.2 |
| No | 451(93.4%) | 252(92.0%) | 199(95.2%) | |  |
| Yes | 32(6.6%) | 22(8.0%) | 10(4.8%) | |  |
| **Weekly family face-to-face communication** | 20 (14,24) | 20 (14,25) | 19 (12,23) | | **0.035** |
| **Weekly online communication with friends** | 13 (5,21) | 12 (6,20) | 14 (5,21) | | 0.3 |
| **Age** | 44 (37,50) | 46 (39,51) | 40 (33,48) | | **<0.001** |
| **Religious belief** |  |  |  | | **0.009** |
| No | 357(73.9%) | 215(78.5%) | 142(67.9%) | |  |
| Yes | 126(26.1%) | 59(21.5%) | 67(32.1%) | |  |
| **SWB** |  |  |  | | 0.3 |
| Low | 247(51.1%) | 135(49.3%) | 112(53.6%) | |  |
| High | 236(48.9%) | 139(50.7%) | 97(46.4%) | |  |
| ^1^Categorical variables are presented as number of participants (%); numeric variables are presented as the Median (25%,75%) | | | | |  |
| ^2^Pearson's Chi-squared test; Wilcoxon rank sum test; Fisher's exact test | | | | |  |

Supplementary Table 3 Analysis of differences between the test set and training set

| **Characteristic** | **test**, N = 96^1^ | **train**, N = 387^1^ | **p-value**^2^ |
| --- | --- | --- | --- |
| **Gender** |  |  | 0.6 |
| Male | 52(54.2%) | 222(57.4%) |  |
| Female | 44(45.8%) | 165(42.6%) |  |
| **Ethnic group** |  |  | 0.3 |
| Han | 40(41.7%) | 185(47.8%) |  |
| Others | 56(58.3%) | 202(52.2%) |  |
| **Location of registered residence** |  |  | 0.2 |
| Rural | 61(63.5%) | 219(56.6%) |  |
| Urban | 35(36.5%) | 168(43.4%) |  |
| **Marital status** |  |  | 0.7 |
| Not married | 8(8.3%) | 20(5.2%) |  |
| Married | 83(86.5%) | 344(88.9%) |  |
| Divorced | 3(3.1%) | 13(3.4%) |  |
| Widowed | 2(2.1%) | 10(2.6%) |  |
| **Education** |  |  | 0.3 |
| Elementary school or lower | 17(17.7%) | 57(14.7%) |  |
| Middle school | 24(25.0%) | 135(34.9%) |  |
| High school or vocational school | 41(42.7%) | 135(34.9%) |  |
| College or higher | 14(14.6%) | 60(15.5%) |  |
| **Father's education** |  |  | >0.9 |
| Elementary school or lower | 57(59.4%) | 226(58.4%) |  |
| Middle school | 31(32.3%) | 128(33.1%) |  |
| High school or vocational school | 8(8.3%) | 31(8.0%) |  |
| College or higher | 0(0.0%) | 1(0.3%) |  |
| Other | 0(0.0%) | 1(0.3%) |  |
| **Mother's education** |  |  | 0.6 |
| Elementary school or lower | 68(70.8%) | 278(71.8%) |  |
| Middle school | 24(25.0%) | 93(24.0%) |  |
| College or higher | 1(1.0%) | 1(0.3%) |  |
| Other | 3(3.1%) | 15(3.9%) |  |
| **Income** | 60,000 (24,000,72,000) | 69,000 (23,500,100,000) | **0.022** |
| **Frequency of health issues affecting daily activities** |  |  | >0.9 |
| Always | 0(0.0%) | 2(0.5%) |  |
| Often | 3(3.1%) | 12(3.1%) |  |
| Sometimes | 34(35.4%) | 137(35.4%) |  |
| Rarely | 47(49.0%) | 193(49.9%) |  |
| Never | 12(12.5%) | 43(11.1%) |  |
| **Frequency of physical exercise** |  |  | 0.4 |
| Never | 18(18.8%) | 48(12.4%) |  |
| A few times a year or less | 31(32.3%) | 123(31.8%) |  |
| A few times a month | 30(31.3%) | 147(38.0%) |  |
| Several times a week | 13(13.5%) | 45(11.6%) |  |
| Daily | 4(4.2%) | 24(6.2%) |  |
| **Frequency of friend gatherings** |  |  | 0.3 |
| Never | 4(4.2%) | 12(3.1%) |  |
| A few times a year or less | 41(42.7%) | 206(53.2%) |  |
| A few times a month | 46(47.9%) | 154(39.8%) |  |
| Several times a week | 4(4.2%) | 13(3.4%) |  |
| Daily | 1(1.0%) | 2(0.5%) |  |
| **Frequency of relative gatherings** |  |  | 0.6 |
| Never | 10(10.4%) | 31(8.0%) |  |
| A few times a year or less | 74(77.1%) | 306(79.1%) |  |
| A few times a month | 11(11.5%) | 47(12.1%) |  |
| Several times a week | 1(1.0%) | 1(0.3%) |  |
| Daily | 0(0.0%) | 2(0.5%) |  |
| **Frequency of social activities** |  |  | 0.6 |
| Never | 1(1.0%) | 5(1.3%) |  |
| Rarely | 22(22.9%) | 112(28.9%) |  |
| Sometimes | 50(52.1%) | 165(42.6%) |  |
| Often | 21(21.9%) | 94(24.3%) |  |
| Always | 2(2.1%) | 11(2.8%) |  |
| **Frequency of neighbor social activities** |  |  | >0.9 |
| Never | 4(4.2%) | 17(4.4%) |  |
| Rarely | 24(25.0%) | 111(28.7%) |  |
| Sometimes | 55(57.3%) | 202(52.2%) |  |
| Often | 10(10.4%) | 45(11.6%) |  |
| Always | 3(3.1%) | 12(3.1%) |  |
| **Frequency of friend social activities** |  |  | 0.6 |
| Never | 1(1.0%) | 6(1.6%) |  |
| Rarely | 36(37.5%) | 155(40.1%) |  |
| Sometimes | 45(46.9%) | 149(38.5%) |  |
| Often | 12(12.5%) | 69(17.8%) |  |
| Always | 2(2.1%) | 8(2.1%) |  |
| **Trust in society** |  |  | 0.4 |
| Strongly disagree | 3(3.1%) | 16(4.1%) |  |
| Comparatively disagree | 8(8.3%) | 44(11.4%) |  |
| Neutrality | 25(26.0%) | 93(24.0%) |  |
| Relatively agree | 58(60.4%) | 208(53.7%) |  |
| Couldn't agree more | 2(2.1%) | 26(6.7%) |  |
| **Personal socio-economic status** |  |  | 0.2 |
| Lower class | 9(9.4%) | 38(9.8%) |  |
| Lower middle class | 23(24.0%) | 95(24.5%) |  |
| Middle-ranking | 56(58.3%) | 186(48.1%) |  |
| Upper-middle class | 7(7.3%) | 63(16.3%) |  |
| Upper class | 1(1.0%) | 5(1.3%) |  |
| **Family economic status** |  |  | 0.2 |
| Well below average level | 7(7.3%) | 30(7.8%) |  |
| Below average level | 24(25.0%) | 103(26.6%) |  |
| Average level | 56(58.3%) | 182(47.0%) |  |
| Above average level | 9(9.4%) | 65(16.8%) |  |
| Well above average level | 0(0.0%) | 7(1.8%) |  |
| **Number of properties owned** |  |  | 0.2 |
| 0 | 6(6.3%) | 11(2.8%) |  |
| 1 | 78(81.3%) | 294(76.0%) |  |
| 2 | 12(12.5%) | 72(18.6%) |  |
| 3 | 0(0.0%) | 7(1.8%) |  |
| 4 | 0(0.0%) | 3(0.8%) |  |
| **Household car ownership** |  |  | 0.5 |
| Yes | 46(47.9%) | 200(51.7%) |  |
| No | 50(52.1%) | 187(48.3%) |  |
| **Family investment activities** |  |  | **0.046** |
| No | 94(97.9%) | 357(92.2%) |  |
| Yes | 2(2.1%) | 30(7.8%) |  |
| **Weekly family face-to-face communication** | 18 (12,23) | 20 (14,25) | 0.060 |
| **Weekly online communication with friends** | 12 (5,20) | 13 (5,21) | 0.5 |
| **Age** | 45 (34,49) | 44 (37,50) | 0.7 |
| **Religious belief** |  |  | >0.9 |
| No | 71(74.0%) | 286(73.9%) |  |
| Yes | 25(26.0%) | 101(26.1%) |  |
| **Subjective well-being** |  |  | >0.9 |
| Low | 49(51.0%) | 198(51.2%) |  |
| High | 47(49.0%) | 189(48.8%) |  |
| ^1^Categorical variables are presented as number of participants (%); numeric variables are presented as the Median (25%,75%) | | | |
| ^2^Pearson's Chi-squared test; Wilcoxon rank sum test; Fisher's exact test | | | |
