# Supplementary figures and images for: Prediction of subjective well-being level in residents of Dali City: Where modern tourism meets traditional ethnic culture
Source: PLoS One. 2025 Sep 26;20(9):e0332625. doi: 10.1371/journal.pone.0332625 (PMC12469199; doi:10.1371/journal.pone.0332625)

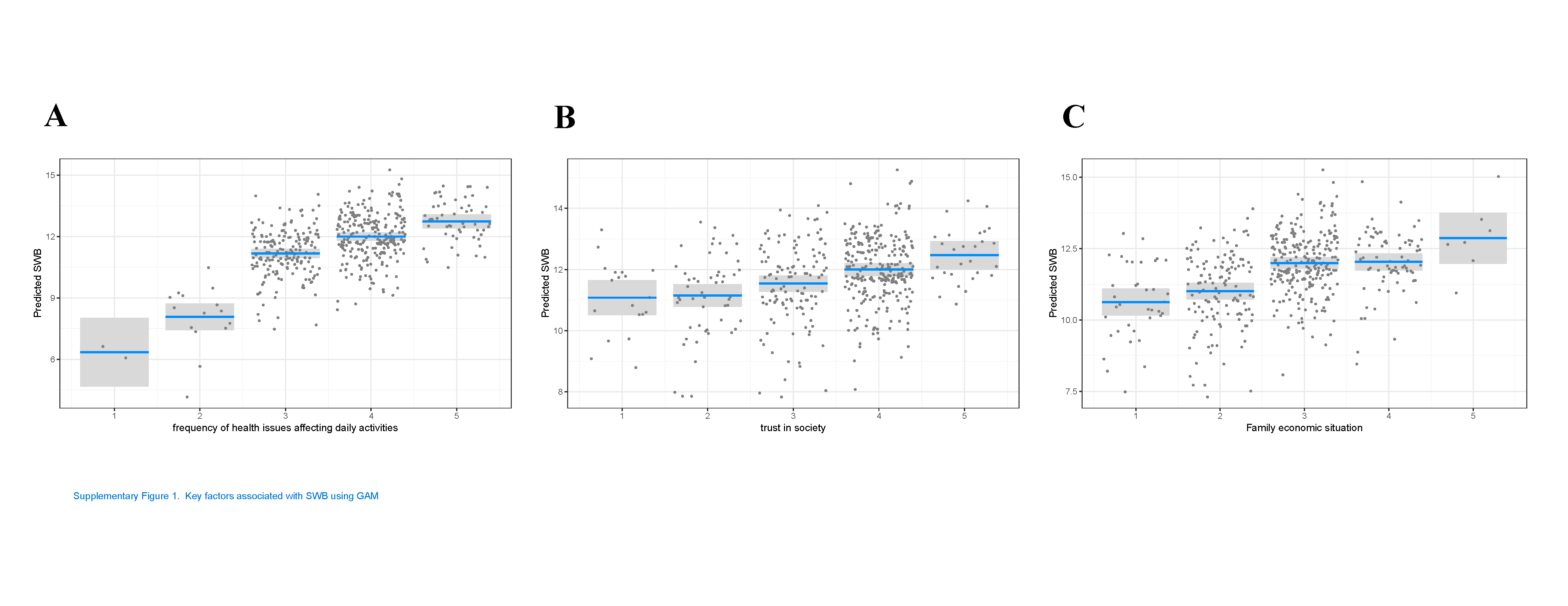

Supplement: S2 File — (TIF) [file pone.0332625.s002.tif]
